# BORA overexpression promotes epithelial–mesenchymal transition and metastasis in ovarian cancer: Unveiling a novel therapeutic target for advanced disease

**DOI:** 10.1002/ctm2.70285

**Published:** 2025-03-28

**Authors:** Marta Barber, Ariadna Boloix, Alfonso Parrilla, Mariana Köber, Laia Avilés‐Domínguez, Nora Ventosa, Lidia del Carmen Ramírez‐Morales, Asunción Perez‐Benavente, Antonio Gil‐Moreno, Eva Colàs, Juan Morote, Miguel F. Segura, Olga Méndez, Anna Santamaria

**Affiliations:** ^1^ Group of Biomedical Research in Urology Vall Hebron Research Institute (VHIR) Universitat Autònoma de Barcelona (UAB) Barcelona Spain; ^2^ Department of Cancer Group of Childhood Cancer and Blood Disorders Vall d'Hebron Research Institute (VHIR) Universitat Autònoma de Barcelona (UAB) Barcelona Spain; ^3^ Institut de Ciència de Materials de Barcelona ICMAB‐CSIC, Campus UAB Bellaterra Spain; ^4^ CIBER de Bioingeniería Biomateriales y Nanomedicina Instituto de Salud Carlos III Bellaterra Spain; ^5^ Group of Biomedical Research in Gynecology Vall Hebron Research Institute (VHIR) Universitat Autònoma de Barcelona (UAB) Barcelona Spain

1

Dear Editor,

We are pleased to present our latest findings, which demonstrate that BORA plays a key role in the metastatic capacity of ovarian cancer (OC) cells by triggering a PLK1‐mediated induction of epithelial‐mesenchymal transition (EMT). Additionally, our research suggests that the inhibition of BORA could offer a novel strategy for improving OC prognosis.

OC is the most lethal gynaecologic malignancy given that most patients are diagnosed at advanced stages, when the disease has already metastasized, and the 5‐year survival rate is below 30%.^1^ The most common and aggressive subtype is high‐grade serous carcinoma (HGSC), which is a highly heterogeneous tumour generally chemoresistant.^2^

Polo‐like kinase 1 (PLK1), a master regulator of mitosis,^3^ is responsible for triggering EMT in various cancers by activating multiple signalling pathways (Figure S1).^4^, ^5^ Although several PLK1 inhibitors have been developed, their antitumour activity against solid tumours is modest, primarily due to poor selectivity and toxicity arising from targeting other PLK family members.^6^, ^7^ Notably, BORA, a specific cofactor of PLK1, has emerged as a potential target for selectively blocking PLK1 activity. BORA activates PLK1 by binding it, causing a conformational change that enables Aurora A to phosphorylate PLK1 at T210, initiating mitotic entry.^3^ We propose that BORA not only activates PLK1 to trigger mitosis but also to induce EMT.

Previous results from our group demonstrated that BORA expression is higher in OC metastatic samples than in paired primary tumours.^8^ In the present study, we confirmed that BORA mRNA expression (GSE73168) was higher in ascitic fluid‐derived OC cells than in the matched primary tumours (Figure S2). Transcriptomic analyses were performed to elucidate BORA mechanism of action. In vivo overexpression of BORA (BORA_OE) in SKOV3 OC xenografts led to an enrichment of genes associated with EMT and migration (Figure 1A,B). Significantly upregulated genes included the main mesenchymal marker N‐cadherin (*CDH2*) and several matrix metalloproteases (e.g., *MMP13*) (Figure 1B). The correlation between BORA mRNA expression and the expression of several metastasis‐associated genes was validated in the ovarian serous cystadenocarcinoma TCGA cohort (2022‐v32) (Figure S3D–G).

**FIGURE 1:**
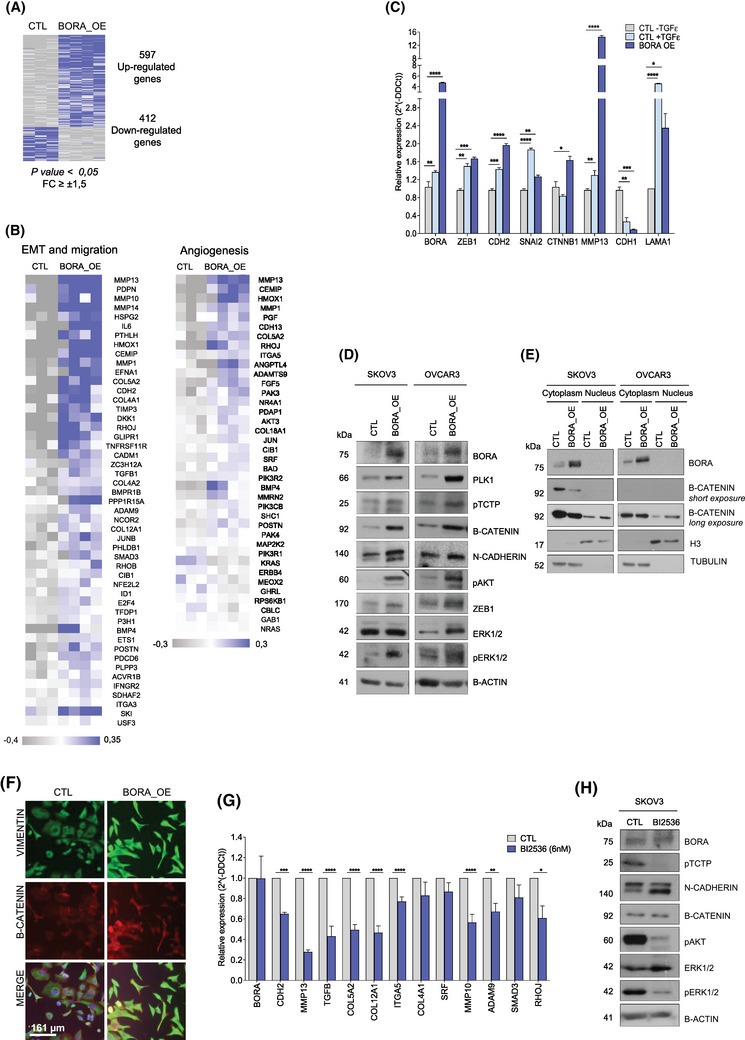
BORA overexpression increases the expression of mesenchymal‐related genes through PLK1 activation. (A) Heat map comparing the transcriptional profile of BORA_OE (*n* = 4) and CTL (*n* = 3) tumours. Cut‐off criteria for deregulated genes were fold change (FC) ≥ 1.5/‐1.5 and *p* < 0.05. Grey, under‐expressed genes; blue, overexpressed genes. (B) Heat map of selected differentially expressed genes involved in EMT, cell migration and angiogenesis, obtained from a gene set enrichment analysis (GSEA). Grey, under‐expressed genes; blue, overexpressed genes. (C) Relative mRNA expression of several EMT‐related genes in SKOV3_BORA_OE cells compared to that in SKOV3_CTL + TGFβ and SKOV3_CTL—TGFβ cells. Gene expression of SKOV3_BORA_OE cells and of SKOV3_CTL + TGFβ cells was relativized to that in SKOV3_CTL—TGFβ cells. mRNA expression values were normalized to that of TBP (TATA‐box binding protein). (D) Immunoblot showing the expression of BORA, PLK1, phospho‐TCTP, and the EMT‐related markers β‐catenin, N‐cadherin, phospho‐AKT, ZEB1, and phospho‐ERK1/2 in SKOV3 and OVCAR3 BORA_OE cells compared to that in CTL cells. β‐Actin was used as loading control. (E) Immunoblot showing the nuclear and cytoplasmic BORA and β‐catenin protein levels at two different exposures (short and long). Histone 3 (H3) and tubulin were used to control equal protein loading for the nuclear and cytoplasmic fractions, respectively. (F) Immunofluorescence staining of Vimentin (green) and β‐catenin (red) in SKOV3_CTL and BORA_OE cells; a higher expression of both proteins is observed in the latter group. Scale bar: 161 µm. (G) mRNA expression levels of several genes involved in metastasis in SKOV3_BORA_OE cells treated with the PLK1 inhibitor BI2536 (6 nM; for 4 h) compared to those in nontreated cells. mRNA expression values were normalized to that of the endogenous control TBP. (H) Immunoblot illustrating the levels of BORA, phospho‐TCTP, N‐cadherin, β‐catenin, phospho‐AKT, and phospho‐ERK1/2 in SKOV3_BORA_OE cells either untreated or treated with BI2536. β‐Actin was used as loading control. *p*‐values were calculated using a two‐way ANOVA test. *p < 0.05; **p < 0.01; ***p < 0.001; ****p < 0.0001.

To confirm whether the gene expression deregulation produced by BORA_OE generated a mesenchymal phenotype, we compared BORA_OE cells with control cells treated with TGFβ, a well‐known EMT inducer. Our results showed that, under both conditions, the mRNA levels of the mesenchymal markers ZEB1, SNAI2, MMP13, and LAMA1 were markedly increased, whereas the expression of the epithelial marker CDH1 was reduced (Figure 1C). At the protein level, BORA_OE increased the levels of N‐cadherin, ZEB1, and the nuclear fraction of β‐catenin (Figure 1D–F). Additionally, BORA_OE increased the phosphorylation of AKT and ERK1/2, two kinases associated with PLK1‐mediated EMT (Figure 1D). Therefore, BORA_OE induces EMT and the acquisition of mesenchymal features. We also confirmed that BORA triggers EMT through PLK1, as treatment with the PLK1 inhibitor BI2536 blocked BORA_OE induced effects (Figure 1G,H).

Attachment of OC cells to the peritoneal wall prior to their invasion into the submesothelial stroma is a key step in OC metastasis. Therefore, we tested the ability of SKOV3 cells to attach to collagen. Our in vitro studies demonstrated that SKOV3_BORA_OE cells had a higher capacity to attach to collagen than control cells, and that BORA_OE multicellular aggregates disaggregated and disseminated through collagen‐coated plates faster than the controls (Figure 2A,B; FigureS3A–D). MMP13 enzymatic activity, which is involved in extracellular matrix degradation, was also higher in BORA_OE cells (Figure S4D). Remarkably, BORA_OE increased both the migration (Figure 2C) and invasive (Figure 2D) capacities of SKOV3 and OVCAR3 cells —which are more epithelial‐like than SKOV3 cells—independently of proliferation (Figure S5). Altogether, BORA_OE may favour OC cell migration, attachment to, and colonization of metastatic sites.

**FIGURE 2:**
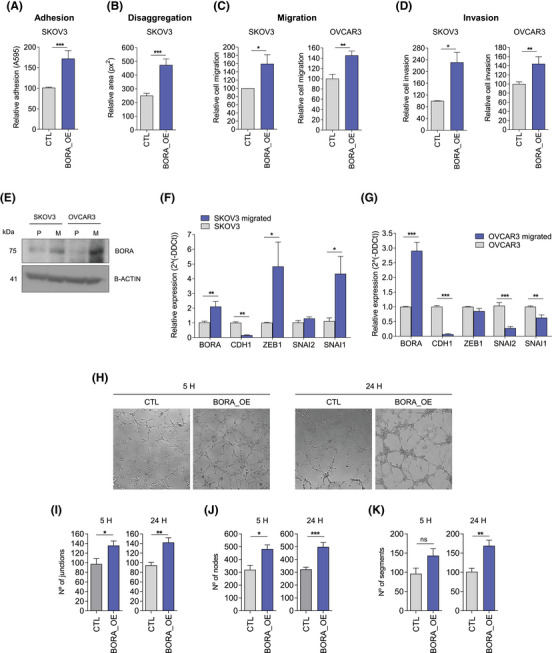
High BORA expression increases metastasis‐related properties of OC cells in vitro. (A) Relative quantification of the adhesion capacity of SKOV3_BORA_OE and CTL cells. The adhesion capacity was calculated by comparing the optical density (at 595 nm), after crystal violet staining, of SKOV3_BORA_OE and CTL adhered cells. (B) Relative quantification of the area (px2) occupied by disaggregated and disseminated SKOV3_BORA_OE and CTL cells. The ratio between the area at 24 and 1 h post‐seeding was calculated for each condition. (C) Relative migration capacities of SKOV3 and OVCAR3 BORA_OE and CTL cells. (D) Relative invasion capacities of SKOV3 and OVCAR3 BORA_OE and CTL cells. BORA_OE cell migration and invasion values were relativized to those of CTL cells. (E) Immunoblot illustrating the expression of BORA in parental (P) and migrated (M) SKOV3 and OVCAR3 cells. β‐actin was used as loading control. (F) Relative mRNA expression of BORA, CDH1, ZEB1, SNAI2, and SNAI1 in migrated SKOV3 cells compared to that in parental SKOV3 cells. (G) Relative mRNA expression of BORA, CDH1, ZEB1, SNAI2, and SNAI1 in migrated OVCAR3 cells compared to that in parental OVCAR3 cells. mRNA expression values were normalized to that of TBP. (H) Representative images of the tube formation assay showing that BORA overexpression favours angiogenesis. (I–K) Number of tube (I) junctions, (J) nodes and (K) segments formed by HUVEC cells at 5 and 24 h post‐seeding with OVCAR8_CTL or OVCAR8_BORA_OE conditioned media. *p*‐values were calculated using a two‐tailed Student's *t*‐test. *p < 0.05; **p < 0.01; ***p < 0.001.

In agreement with the effect of ectopic BORA_OE, high endogenous BORA levels favoured cell migration. After subjecting OVCAR3 and SKOV3 parental cells to five sequential cycles of transwell migration to obtain a highly migratory population (Figure S6), we demonstrated that both OVCAR3 (FC = 2.8) and SKOV3 (FC = 2) migrated cells expressed higher levels of BORA than the parental cells (Figure 2E–G). This was accompanied by an almost undetectable expression of E‐cadherin (*CDH1*) (Figure 2F–G). Additionally, in SKOV3 migrated cells, transcripts encoding EMT regulators, such as ZEB1 and SNAI1, were upregulated (Figure 2F). Controversially, OVCAR3 migrated cells expressed lower levels of SNAI1 and SNAI2 (Figure 2G), suggesting that they had reverted to a more epithelial‐like phenotype. These differences may be due to the fact that OVCAR3 cells exhibit an epithelial morphology under basal conditions, while SKOV3 cells are mesenchymal. As a result, OVCAR3 cells may require stronger and more sustained stimuli to maintain a mesenchymal phenotype.

In our transcriptional analysis, we detected an enrichment of gene sets related to angiogenesis, which is required for metastasis (Figure 1B). This association was also observed in TCGA data (Figure S7). At the functional level, our results showed that endothelial cells (i.e., HUVEC) cultured in conditioned medium derived from OVCAR8 cells overexpressing BORA (OVCAR8_BORA_OE) formed more tubes than those seeded in medium from control cells (Figure 2H–K).

To assess whether BORA_OE confers enhanced metastatic capacity to OC cells, we injected OVCAR3_CTL or OVCAR3_BORA_OE cells into immunosuppressed NOD/SCID mice. Remarkably, all mice in the BORA_OE group developed detectable metastases with high bioluminescence intensity, whereas only 3 of the CTL group developed metastases. This confirmed that BORA_OE increases the capacity of OC cells to form intraperitoneal metastatic lesions (Figure 3A–F).

**FIGURE 3:**
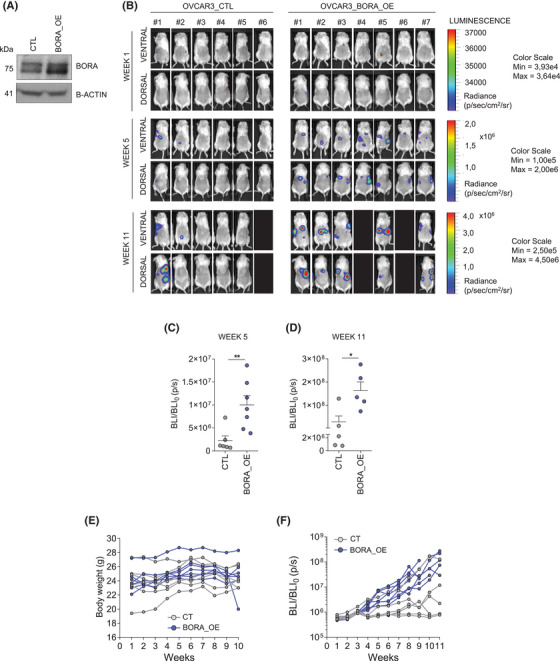
BORA overexpression enhances the formation of intraperitoneal metastatic lesions in vivo. (A). Immunoblot of BORA expression in OVCAR3_CTL and BORA_OE cells. β‐actin was used as loading control. (B). Luciferase activity in mice at 1, 5, and 11 weeks post‐injection. Black boxes represent dead mice. The luminescence scale is shown on the right. (C–D) Scatter dot plots showing average tumour bioluminescence ± SEM (photons/seconds; p/s) in mice at (C) 5 and (D) 11 weeks post‐injection. (E) Individual mice weight fluctuation determination. (F) Individual tumour bioluminescence values (p/s). *p*‐values were calculated using a two‐tailed Student's *t*‐test. *p < 0.05; **p < 0.01.

Finally, we tested BORA silencing as a potential therapeutic approach against OC by conjugating a siRNA against BORA (siBORA) with Quatsomes (QS), a novel type of non‐liposomal lipid‐based nanovesicles.^10^ The siBORA‐QS complexes efficiently reduced BORA protein levels, increased PARP cleavage (Figure 4A), and significantly decreased cell proliferation (Figure 4B), suggesting the induction of apoptosis. Importantly, when this approach was tested on multicellular aggregates generated from ascitic fluid‐derived OC cells, a significant reduction in cell viability was observed (Figure 4C,D). Finally, the combination of BORA silencing with current therapies, such as cisplatin (Figure 4E) and paclitaxel (Figure 4F), enhanced the efficacy of single treatments in reducing cell proliferation. Therefore, targeting BORA in combination with the standard of care may represent a novel strategy for improving OC prognosis.

**FIGURE 4:**
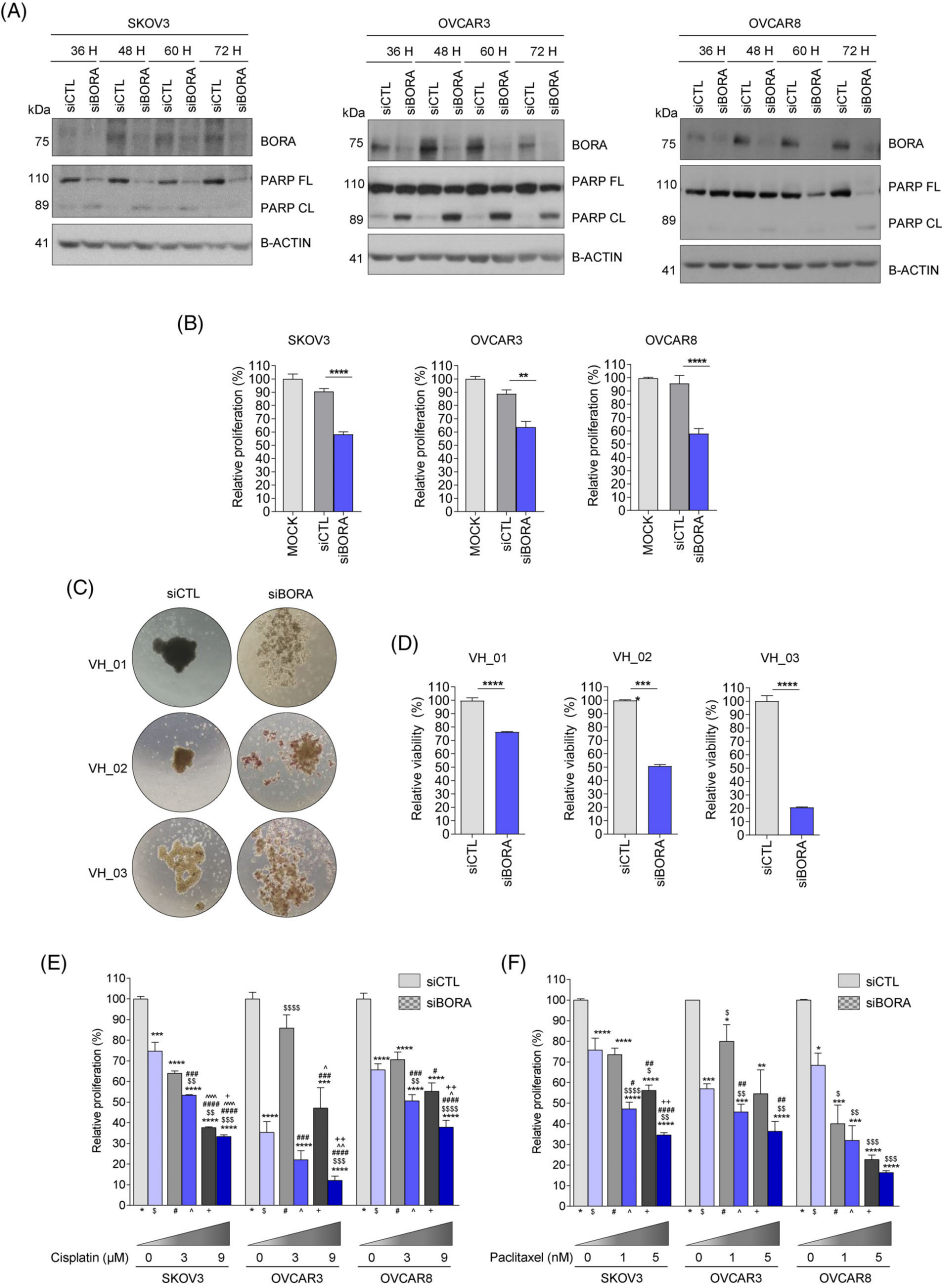
BORA silencing reduces ovarian cancer cell proliferation and survival. (A) Immunoblot showing the capacity of siBORA‐QS complexes to decrease BORA expression and induce the cleavage of PARP (PARP CL) in three different cell lines at 36, 48, 60, and 72 h post‐transfection. BORA, full length PARP (PARP FL), and PARP CL expression levels are shown for each condition. β‐actin was used as loading control. (B) Proliferation of SKOV3, OVCAR3, and OVCAR8 cells transfected for 96 h with siBORA or siCTL. Proliferation was relativized to that of non‐transfected cells (MOCK). (C) Representative images of three patient‐derived multicellular aggregates at 96 h post‐transfection with siCTL‐QS or siBORA‐QS complexes. Scale bar: 161 µm. (D) Cell viability of OC patient‐derived multicellular aggregates measured at 96 h post‐transfection. (E) SKOV3, OVCAR3, and OVCAR8 cell proliferation measured at 144 h post‐transfection with siRNAs‐QS and 72 h post‐cisplatin treatment. (F) SKOV3, OVCAR3, and OVCAR8 cell proliferation measured at 144 h post‐transfection with siRNAs‐QS and 72 h post‐paclitaxel treatment. For A and D, *p*‐values were calculated using a two‐tailed Student's *t*‐test. *p < 0.05; **p < 0.01; ***p < 0.001; ****p < 0.0001. For E and F, *p‐*values were calculated using a two‐way ANOVA test. * compares siCTL + 0 nM vs. all other conditions; # compares siBORA + 0 nM vs. all other conditions; $ compares siCTL + 1 nM vs. all other conditions, ^ compares siBORA + 1 nM vs. all other conditions; and + compares siCTL + 5 nM vs. all other conditions *,$.#,^.+*p* < 0.05; **,$$.##,^^.++*p* < 0.01; ***,$$$.###,^^^.+++ *p* < 0.001; ****,$$$$.####,^^^^.++++*p* < 0.0001.

In summary, our findings suggest that BORA contributes to OC dissemination by triggering EMT via PLK1. Furthermore, RNA‐mediated BORA silencing may reduce OC metastasis and improve the therapeutic effects of current treatments.

## AUTHORS CONTRIBUTORS

Marta Barber and Anna Santamaria designed the study. Marta Barber, Ariadna Boloix and Alfonso Parrilla carried out the experiments and analyzed data. Ariadna Boloix, Mariana Köber, Laia Avilés‐Domínguez and Nora Ventosa provided the nanovesicles used in the study and participated in the critical revision of the manuscript. Lidia del Carmen Ramírez‐Morales participated in the revision of the manuscript. Asunción Perez‐Benavente, Antonio Gil‐Moreno and Eva Colàs provided patient‐derived samples. Miguel F. Segura and Olga Méndez provided intellectual support for result interpretation and critical revision. Juan Morote and Anna Santamaria secured the funding of this study. Marta Barber and Olga Méndez wrote the manuscript. All the authors read and approved the final manuscript.

## FUNDING INFORMATION

This work was supported in part by grants from Instituto de Salud Carlos III (PI15/00238) to Anna Santamaria. This work was funded by the Instituto de Salud Carlos III through the projects (PI23/01144), (ICI21000/76), (FORT23/00034) (Co‐funded by the European Regional Development Fund/European Social Fund; “A way to make Europe”/“Investing in your future”) to Miguel F. Segura. Marta Barber was supported by predoctoral fellowship from AGAUR (2020 FI_B 00058). This work was funded by Ministerio de Ciencia e Innovación (#PID2022‐137332OB‐I00) and ajuts d'Indústria del Coneixement (2021 PROD 00059) to Nora Ventosa.

## CONFLICT OF INTEREST STATEMENT

The authors declare no conflicts of interest.

## ETHICS STATEMENT

All animal experimental procedures were approved by the Vall d'Hebron Hospital Animal Experimentation Ethics Committee (protocol number 03.18). Informed consent was obtained from all patients for the research use of their samples.

## Supporting information



Supporting Information

## Data Availability

The authors confirm that all data supporting the findings of this study are available within the article, supplemental material and the corresponding web servers. Further information from the transcriptomic analysis (CEL files) are available at the GEOarchive repository GSE139244.
