# Supplementary material for: BORA overexpression promotes epithelial–mesenchymal transition and metastasis in ovarian cancer: Unveiling a novel therapeutic target for advanced disease
Source: Clin Transl Med. 2025 Mar 28;15(4):e70285. doi: 10.1002/ctm2.70285 (PMC11950690; doi:10.1002/ctm2.70285)
Supplement: Supplementary file 1 — Supporting Information [file CTM2-15-e70285-s001.pdf]

# BORA overexpression promotes epithelial-mesenchymal transition and metastasis in ovarian cancer: unveiling a novel therapeutic target for advanced disease

## SUPPORTING INFORMATION

### Materials and methods

#### Analysis of OC gene expression data sets

Gene expression and correlation analyses was performed by mining data of the publicly-available transcriptomic datasets GSE73168 and Tumor Ovarian Serous Cystadenocarcinoma TCGA cohort (2022-v32) using the R2 Genomics website (http://r2.amc.nl).

#### Cell lines and primary cultures

SKOV3 and OVCAR3 OC cell lines were acquired from American Type Culture Collection (ATCC); HEK-293T were obtained from Dr. Erich A. Nigg's lab (Basel University, Switzerland), and OVCAR8 cells were a generous gift from Dr. Dan Peer (Tel Aviv University, Israel). Table S1 summarizes the three OC cell lines' characteristics. The human endothelial cell line HUVEC was a generous gift from Dr. Laura Soucek (Vall d'Hebron Oncology Institute, Spain). OC cells were cultured in RPMI medium (Biowest); HEK-293T cells in High glucose Dulbecco's modified Eagle's medium (Biowest), and HUVEC cells in F-12K medium (ThermoFisher). Media were supplemented with 2 mM L-glutamine (Biowest), 1% penicillin-streptomycin solution (Biowest), 1% MEM non-essential amino acids (Biowest), 1% sodium pyruvate (Biowest) and 10% heat-inactivated fetal bovine serum (FBS), except for OVCAR3 cells where 20% of FBS was used. F-12K medium was also supplemented with 0.1 mg/mL heparin solution (Sigma-Aldrich) and 30 µg/mL Endothelial Cell Growth Supplement (Fisher Scientific). Cells were regularly tested for mycoplasma contamination and stored in liquid nitrogen. For patient-derived primary cultures, ascitic fluid was collected during surgery after prior consent of the patient, mixed (1:1) with Mixed medium (50% MCDB109, 50% M-199 (Sigma-Aldrich)) and seeded in standard culture flasks. After a week, culture media was replaced with fresh media to remove blood and floating cells. Primary cells were used from passages 2 to 6. All cells were maintained at 37ºC in a saturated atmosphere of 95% air and 5% $CO_2$.

#### Real-Time Quantitative PCR

RNA from tumor tissue or from cell lines was extracted using the miRNeasy Mini Kit (Qiagen) following the manufacturer's instructions. In the case of frozen tumor tissue, first tumor pieces of 2 to 5 mm of diameter were chopped on dried ice and then homogenized using FasPrep 24 Matrix tubes (MP Biomedicals) with 700 µL of QIAzol lysis reagent in the FasPrep-24 instrument. Next, homogenates were centrifugated, supernatants were transferred into collection tubes, and 140 µL of chloroform were added to each tube, to finally proceed with the extraction following the standard protocol of the kit. For RNA retrotranscription 1 µg of total RNA was retrotranscribed into cDNA using the SuperScript III reverse transcriptase (Invitrogen), following the manufacturer's protocol. Real-time quantitative PCR was performed using PowerUP SYBER Green Master Mix

(Applied Biosystems) in the LighCycler 480 System (Roche). Primer sequences are listed in Table S2. Relative quantification of gene expression was performed with the $2^{(-\Delta\Delta Ct)}$ method (Livak KJ, et al. 2001).

**Western blot**

Total protein extracts were obtained in 1x RIPA buffer supplemented with 1x EDTA-free complete protease inhibitor (Roche) and phosphatase cocktail inhibitors (Sigma). To isolate nuclear and cytoplasmic cellular subfractions, the NE-PER™ Nuclear and Cytoplasmic extraction Kit (Thermo Scientific) was used following the manufacturer's instructions. To obtain protein extracts from mice tumor xenografts, tumor pieces of 2 to 5 mm diameter were chopped in 200 to 500 µL of RIPA buffer and disrupted with a tissue homogenizer. Proteins (50-100 µg total protein extract, 15 µg nuclear subfraction or 25 µg cytoplasmic subfraction) were resolved in NuPAGE 4-12% Bis-Tris gels and transferred onto polyvinylidene difluoride (PVDF) membranes and blocked for 1 h with 5% nonfat milk. Membranes were probed overnight at 4 ºC with the indicated antibodies. Protein expression was normalized to β-actin (total protein extracts), α-tubulin (cytoplasmic subfraction) or histone H3 (nuclear subfraction).

**Immunofluorescence**

Cells were grown on collagen-coated glass coverslips, fixed with 4% paraformaldehyde, permeabilized with 1x TBS with 0.5% Triton™ X-100 and blocked with 2% bovine serum albumin (BSA). Samples were incubated for 1 h at RT with the indicated primary antibody, and after washed, with the secondary antibody for another hour. Finally, cells were mounted on a glass slide in Prolong Damond Antifade mountant with DAPI (Thermo Fisher). Images were obtained using the fluorescence microscope Olympus FSX100 (Olympus).

**Plasmids, lentiviral production and generation of stable cell lines**

For inducible BORA overexpression, the coding sequence of BORA was subcloned from pENTR/D-TOPO vector (Dr. Erich Nigg's lab, Basel University, Switzerland) into the pINDUCER20 vector (Addgene) using Gateway LR Clonase II (Invitrogen) following the manufacturer's instructions. Lentiviral particles were produced in HEK-293T cells using 2nd generation lentiviral plasmids (envelope plasmid MD2.G and packaging plasmid psPAX2 (Addgene). Vectors used are listed in Table S3. OC cells were transduced with fresh viral supernatant plus 5-8 µg/mL of polybrene. For selection of pINDUCER_BORA transduced cells, cells were treated with 0.5-1 mg/mL of geneticin. BORA overexpression was induced with 0.1 µg/mL doxycycline.

**Production of Quatsomes nanovesicles for siRNA delivery**

The Quatsomes used here were developed by A. Boloix et al., and their production has been reported in detail previously (Grimaldi N, et al. 2015). In short, QS were prepared using the DELOS-SUSP (Depressurization of an Expanded Liquid Organic Solution-Suspension) technology (Cabrera I, et al. 2014; Ventosa N, et al. 2006; Boloix A, et al. 2022): First a solution of 0.065 M of Cholesteryl N-(2-dimethylaminoethyl) carbamate (DC-Chol) in ethanol was added to a high-pressure vessel, which was then pressurized upon the introduction of $CO_2$ to create an expanded organic solution. Afterwards, the $CO_2$-expanded solution was depressurized over the aqueous phase consisting of 0.008 M of Benzyldimethyltetradecylammonium Chloride (MKC) in Milli-Q $H_2O$. One week after vesicle production by DELOS-SUSP, the QS were purified by tangential flow filtration using the

KrosFlo® Research Iii TFF System (Spectrum Labs, Repligen Corporation; Waltham, Massachusetts, USA), removing the remaining ethanol and free surfactant (Boloix A, et al. 2022).

**Gene silencing**

For loss of function experiments, a custom siRNA duplex against BORA (siBORA) and a siRNA negative control (siCTL) with [dT][dT] overhangs were purchased from Sigma-Aldrich, based on validated target sequences extracted from previous work of our group (Parrilla A, et al. 2020). siRNA sequences are listed in Table S4. For lipofectamine transfection, 50 nM (final concentration) of siRNA and of Lipofectamine 2000 were mixed in OptiMEM serum-free media, and cells were reversely transfected. For Quatsomes (QS) transfection, siRNA were mixed in a N/P ratio 1:1.25 (siRNA:QS, assuming only the positive charge of the MKC surfactant and not the pH-sensitive positive charges of DC-Chol) with QS at 1.99 mg/mL stock concentration and diluted in PBS 1X, reaching a final concentration in the complex of 0.22 mg/mL of QS and 2.5µM of siRNA (Boloix A, et al. 2022). Cells seeded with the indicated medium without antibiotics were transfected with the mix at a final concentration of 4.35 µg/mL QS and 50 nM siRNA.

**Proliferation growth assays**

To determine the effect of BORA overexpression on OC cell proliferation, OC cells were seeded at a density of $1 \cdot 10^5$ cells into 35 mm-dish with media containing 0.1 µg/mL doxycycline. Cells were counted using trypan blue and reseeded at days 4, 8 and 10. The number of cells was normalized to the number of cells at day 0 ($1 \cdot 10^5$). To determine the effects of BORA silencing, OC cells were seeded at a density of $2 \cdot 10^4$ cells per well on 24-well plates, transfected with the indicated siRNA-QS complex and, at the indicated time point, fixed with formaldehyde 4% solution and stored at 4 ºC in PBS. Cells were stained with 0.5% crystal violet, crystals were dissolved with 15% acetic acid and optical density was read at 590 nm. The effect of siRNA-QS transfection on proliferation was determined by relativizing to the proliferation of non-transfected cells (MOCK). For drug combination studies, OC cells were seeded at a density of $2 \cdot 10^4$ cells per well on 24-well plates and reverse-transfected with QS-siRNA complexes. 72 h after transfection, cells were treated with 3-9 µM cisplatin, or 1-5 nM of paclitaxel for additional 72 h. At the end of the experiment, cells were fixed with 4% paraformaldehyde and stained with 0.5% crystal violet. Finally, crystals were dissolved with 15% acetic acid, and optical density was read at 590 nm.

**Cell viability assays of 3D-cultured patient-derived ascitic cells**

For cell viability assays in 3D patient-derived spheroids, cells derived from patient ascitic fluid were isolated and seeded in anchorage-independent conditions. Thus, cells were grown in mixed medium (1:1-MCDB105:M199) supplemented with 2mM L-Glutamine, 1X B27 vitamin, 20 ng/mL EGF, and 20 ng/mL FGF. For MTS assay, $4 \cdot 10^4$ cells were seeded in non-adherent 12-well plates coated with 0.5% agarose. Simultaneously, cells were transfected with QS-siRNA complexes and 96 h post-transfection cells were disaggregated with 0.5 mL of 1X StemPro Accutase Cell Dissociation Reagent (Thermo Fisher) and PMS:MTS (1:20) mixture was added 1:10 to each well containing 100 µL of disaggregated cells. Finally, optical density was measured at 2-5 h at 490 nm.

**Migration and invasion assays**

For migration assays, $1 \cdot 10^5$ cells suspended in serum-free medium were loaded into the upper chamber of 8.0 µm pore size inserts (Corning). Serum gradient was generated by adding medium with 10% FBS in the lower compartment. Cells were allowed to migrate for 4h (SKOV3 cells) or for 24 h (OVCAR3 cells). For invasion assays, inserts were previously coated with 20 µL of matrigel (Corning) diluted 1:3 in PBS. At the indicated times, cells were fixed with 4% formaldehyde. Cells from the upper part of the membrane were removed with a cotton swab. Migrated and invaded cells were stained with 1µl/mL Hoechst (Invitrogen) and counted.
To select those cells with the highest migratory capacity within a cell population, considering the inherent heterogeneity of cell lines, $1 \cdot 10^5$ SKOV3 or OVCAR3 cells were loaded into the upper chamber of an insert and let them to migrate for 24 h. Then, those cells that migrated to the bottom of the well were collected and expanded, to be subsequently loaded again into a new insert. This cycle of migration was repeated 5 times (Figure S6).

**Disaggregation and spreading assays**

For disaggregation and spreading assays, SKOV3 cells were forced to form multicellular aggregates using the hanging drop method. Briefly, $1 \cdot 10^5$ cells were suspended in 1 mL of media, then drops were formed by hanging 20 µL of the cellular suspension in the cover of a 10 cm plate. After 48 h, drops were collected and one drop per well was seeded in a collagen coated plate. Multicellular aggregates' disaggregation and spreading was monitored by photographing each well at the indicated time point. Multicellular aggregates dissemination was determined by measuring the total area of the multicellular aggregates in each well and then calculating the ratio between the area at the desired time point and at 1 h post-seeding.

**Cell adhesion assay**

For adhesion assays, $3 \cdot 10^3$ cells were seeded per well in type I collagen coated 96-well plates. At the indicated times, cells were fixed with 4% paraformaldehyde solution, stained with 0.5% crystal violet. Crystals were dissolved with 15% acetic acid and optical density was read at 590 nm. Adhesion percentage was calculated by normalizing the absorbance values of each condition against the values of their respective controls on each experiment.

**MMP13 enzymatic activity assay**

Cell culture medium from BORA_OE and CTL cells was collected and stored at -80 ºC until use. Afterwards, MMP13 enzymatic activity was measured using SenosLyte Plus 520 MMP-13 Assay Kit (AnaSpec) following the manufacturer's instructions.

**Mouse Xenografts**

All animal experimental procedures were approved by the Vall d'Hebron Hospital Animal Experimentation Ethics Committee (protocol number 03.18). For subcutaneous xenografts, $2 \cdot 10^6$ SKOV3_BORA_OE or SKOV3_CTL cells were injected into the flank of seven-week old female NMRI nude mice (n=7/group). Tumors were measured every 2-3 days with an electronic caliper. At the end of the experiment, mice were euthanized and tumors were extracted and frozen in liquid nitrogen for further molecular analysis. For intraperitoneal (IP) xenografts, $3 \cdot 10^6$ OVCAR3_BORA_OE or OVCAR3_CTL cells transduced with Firefly luciferase were injected

intraperitoneally in seven-week old female NOD/SCID mice (n=6 CTL/7 BORA_OE). Metastasis growth was followed by *in vivo* bioluminescence imaging once a week and quantified with the Living Image Software. In all experiments, mice welfare was monitored by measuring their body weight twice a week.

**Gene expression analysis**

Total RNA was extracted from control (CTL, n=3) or BORA-overexpressing (BORA_OE, n=4) SKOV3 xenografted tumors. The quality and concentration of RNA was analyzed by RNA NanoChip Bioanalyzer and 200 ng of RNA were hybridized onto Human Clariom$^{TM}$ S assay microarray platform (Affymetrics) at Vall d'Hebron Research Institute (VHIR) genomic facility. Raw data was analyzed with the Expression Console and the Transcriptome Analysis Console (Affymetrix). Functional annotation of the differentially expressed genes was performed using the Gene Set Enrichment Analysis (GSEA) databases. Publicly available collections of gene sets were extracted from the Molecular Signatures Database (MSigDB). *P-value* < 0.05 or FDR < 0.5 were chosen as the cut-off criteria to determine the significantly enriched gene sets. CEL files are available at the GEOarchive repository GSE139244.

**Statistical analysis**

Statistical differences were calculated by two-sided unpaired Student's *t*-test, One-way ANOVA test or Two-way ANOVA test using GraphPad Prism 8.0 (GraphPad Prism Software). Correlation analyses were performed by Pearson's test. Unless indicated, mean ± SEM values are the average of three independent experiments. Data was considered significant as follows: *p< 0.05; **p< 0.01; ***p< 0.001.

**<u>Supplementary references</u>**


- Basu P, Mukhopadhyay A, Konishi I. Targeted therapy for gynecologic cancers: Toward the era of precision medicine. Int J Gynaecol Obstet. 2018;143 Suppl 2:131-136.
- Bruinsma W, Macurek L, Freire R, Lindqvist A, Medema RH. Bora and Aurora-A continue to activate Plk1 in mitosis. J Cell Sci. 2014;127(4):801-11.
- Cabrera I, Elizondo E, Esteban O, et al. Multifunctional nanovesicle-bioactive conjugates prepared by a one-step scalable method using CO2-expanded solvents. Nano Lett 2013;13(8):3766-3774.
- Cai XP, Chen LD, Song HB, et al. PLK1 promotes epithelial-mesenchymal transition and metastasis of gastric carcinoma cells. Am J Transl Res 2016;8(10):4172-4183.
- Cheng S, Peng T, Zhu X, et al. BORA regulates cell proliferation and migration in bladder cancer. Cancer Cell Int 2020;20:290.
- Chiappa M, Petrella S, Damia G, et al. Present and Future Perspective on PLK1 Inhibition in Cancer Treatment. Front Oncol 2022;12:903016.
- Grimaldi N, Andrade F, Segovia N, et al. Lipid-based nanovesicles for nanomedicine. Chem Soc Rev 2016;45(23):6520-6545.
- Heredia-Soto V, López-Guerrero JA, Redondo A, Mendiola M. The hallmarks of ovarian cancer: Focus on angiogenesis and micro-environment and new models for their characterization. EJC Suppl 2020;15:49-55.

- Huang Y, Hong W, Wei X. The molecular mechanisms and therapeutic strategies of EMT in tumor progression and metastasis. J Hematol Oncol 2022;15(1):129.
- Jang HR, Shin SB, Kim CH, et al. PLK1/vimentin signaling facilitates immune escape by recruiting Smad2/3 to PD-L1 promoter in metastatic lung adenocarcinoma. Cell Death Differ. 2021;28(9):2745-2764.
- Kim DE, Shin SB, Kim CH, Kim Y Bin, Oh HJ, Yim H. PLK1-mediated phosphorylation of β-catenin enhances its stability and transcriptional activity for extracellular matrix remodeling in metastatic NSCLC. Theranostics. 2023;13(3):1198-1216.
- Korns J, Liu X, Takiar V. A review of Plks: Thinking outside the (polo) box. Mol Carcinog 2022;61(2):254-263.
- Li S, Pritchard DM, Yu LG. Regulation and Function of Matrix Metalloproteinase-13 in Cancer Progression and Metastasis. Cancers 2022;14(13):3263.
- Livak KJ, Schmittgen TD. Analysis of Relative Gene Expression Data Using Real-Time Quantitative PCR and the 2−ΔΔCT Method. Methods 2001;25(4)402-408.
- Macůrek L, Lindqvist A, Lim D, et al. Polo-like kinase-1 is activated by aurora A to promote checkpoint recovery. Nature 2008;455(7209):119-23.
- Nguyen VHL, Hough R, Bernaudo S, Peng C. Wnt/β-catenin signalling in ovarian cancer: Insights into its hyperactivation and function in tumorigenesis. J Ovarian Res 2019;12(1):122.
- Nieto MA, Huang RY, Jackson RA, Thiery JP. EMT: 2016. Cell. 2016;166(1):21-45.
- Noatynska A, Panbianco C, Gotta M. SPAT-1/Bora acts with Polo-like kinase 1 to regulate PAR polarity and cell cycle progression. Developmen. 2010;137(19):3315-25.
- Prat J, D’Angelo E, Espinosa I. Ovarian carcinomas: at least five different diseases with distinct histological features and molecular genetics. Hum Pathol 2018;80:11-27.
- Raab M, Matthess Y, Raab CA, et al. A dimerization-dependent mechanism regulates enzymatic activation and nuclear entry of PLK1. Oncogene 2022; 41(3):372-386.
- Rizki A, Mott JD, Bissell MJ. Polo-like kinase 1 is involved in invasion through extracellular matrix. Cancer Res. 2007;67(23):11106-11110.
- Shin SB, Jang HR, Xu R, Won JY, Yim H. Active PLK1-driven metastasis is amplified by TGF-β signaling that forms a positive feedback loop in non-small cell lung cancer. Oncogene. 2020;39(4):767-785.
- Tavernier N, Noatynska A, Panbianco C, et al. Cdk1 phosphorylates SPAT-1/Bora to trigger PLK-1 activation and drive mitotic entry in C. elegans embryos. J Cell Biol 2015;208(6):661-9.
- Testa U, Petrucci E, Pasquini L, Castelli G, Pelosi E. Ovarian Cancers: Genetic Abnormalities, Tumor Heterogeneity and Progression, Clonal Evolution and Cancer Stem Cells. Medicines 2018;5(1):16.
- Thiery JP, Acloque H, Huang RY, Nieto MA. Epithelial-mesenchymal transitions in development and disease. Cell 2009;139(5):871-90.
- Ventosa N, Veciana J, Sala S, Cano M. Method for obtaining micro- and nano-disperse systems. WO/2006/079889. 2006.
- Vergara D, Merlot B, Lucot JP, et al. Epithelial-mesenchymal transition in ovarian cancer. Cancer Lett 2010;291(1):59-66.
- Wright PE, Dyson HJ. Intrinsically disordered proteins in cellular signalling and regulation. Nat Rev Mol Cell Biol 2015;16(1):18-29.

- Wu J, Ivanov AI, Fisher PB, Fu Z. Polo-like kinase 1 induces epithelial-to-mesenchymal transition and promotes epithelial cell motility by activating CRAF/ERK signaling. Elife. 2016;5:e10734.
- Xu WJ, Zhang S, Yang Y, et al. Efficient inhibition of human colorectal carcinoma growth by RNA interference targeting polo-like kinase 1 in vitro and in vivo. Cancer Biother Radiopharm 2011;26(4):427-436.
- Yousefi M, Dehghani S, Nosrati R, et al. Current insights into the metastasis of epithelial ovarian cancer - hopes and hurdles. Cell Oncol (Dordr) 2020;43(4):515-538.
- Zhang H, Zhang K, Xu Z, et al. MicroRNA-545 suppresses progression of ovarian cancer through mediating PLK1 expression by a direct binding and an indirect regulation involving KDM4B-mediated demethylation. BMC Cancer 2021;21(1):163.
- Zhao CL, Ju JY, Gao W, et al. Downregulation of PLK1 by RNAi attenuates the tumorigenicity of esophageal squamous cell carcinoma cells via promoting apoptosis and inhibiting angiogenesis. Neoplasma 2015;62(5):748-755.

## Supplementary figures

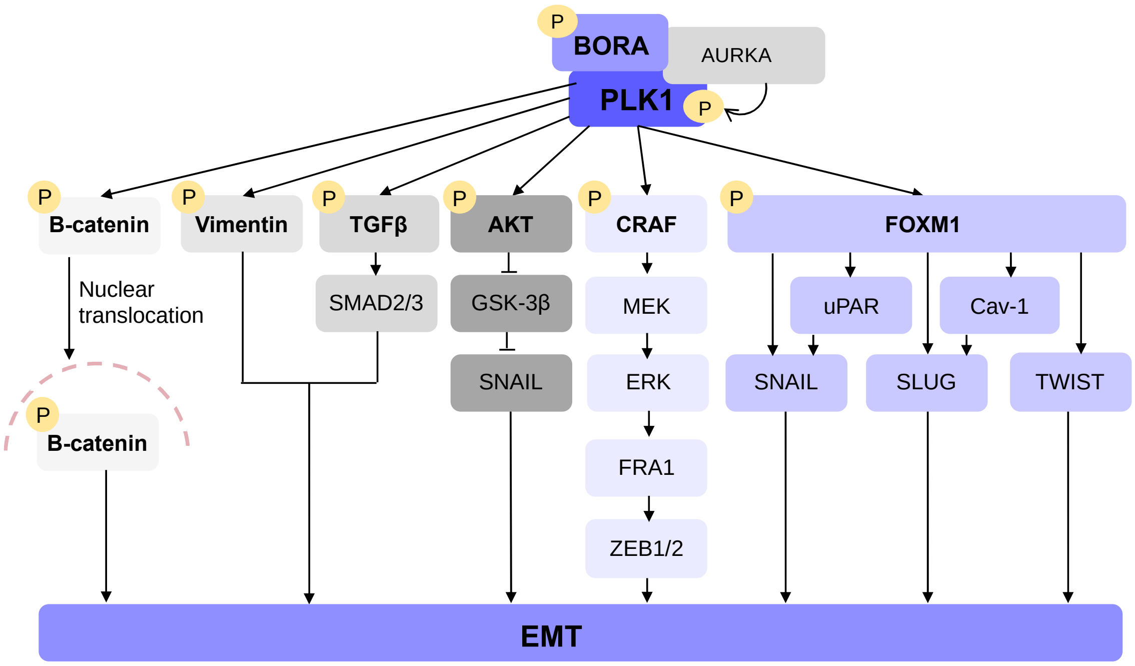


**Supplementary Figure 1. Known signaling pathways through which PLK1 induces EMT.** In prostate cancer cells Wu J, et al. demonstrated that EMT induction requires CRAF phosphorylation by PLK1, leading to the activation of the MEK/ERK cascade which eventually activates ZEB1/2 transcription factors, responsible for the expression of EMT genes (Wu j, et al. 2009). In gastric carcinoma cells, PLK1 overexpression results in downregulation of E-cadherin and upregulation of the mesenchymal markers N-cadherin, SLUG and TWIST, through the phosphorylation of AKT by PLK1 (Cai XP, et al. 2016). In non-small cell lung cancer (NSCLC), PLK1 increases TGFβ expression resulting in the activation of the SMAD pathway and the expression of mesenchymal proteins such SNAIL, SLUG, ZEB1, N-cadherin, IL11 and TNFAIP6 (Shin SB, et al. 2020). Moreover, this pathway is also enhanced by the PLK1-dependent phosphorylation of vimentin, which can then interact with the SMAD2/3 complex triggering its translocation into the nucleus and leading to the expression of EMT-related factors (Jang HR, et al. 2021). In breast cancer, the PLK1-dependent phosphorylation of vimentin results in increased β1 integrin expression in cell surface, promoting cell motility (Rizki A, et al. 2007). PLK1 also triggers EMT indirectly by phosphorylating FOXM1, a promoter of EMT (Chiappa M, et al. 2022). Finally, Kim et al. demonstrated PLK1 phosphorylates β-catenin, which increases β-catenin stability and enhances its nuclear translocation, where promotes the transcription of its target genes, including those responsible of the EMT (Kim DE, et al. 2023).

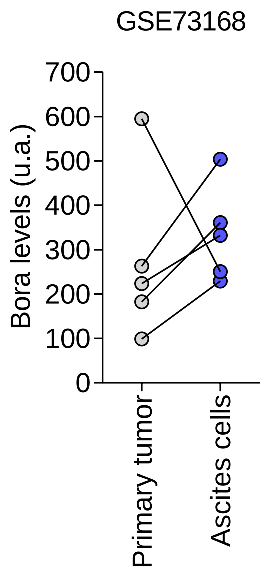


**Supplementary Figure 2.** BORA mRNA expression (arbitrary units; *u.a.*) in five primary OC tumors compared to paired OC cells present in the ascitic fluid (GSE73168).

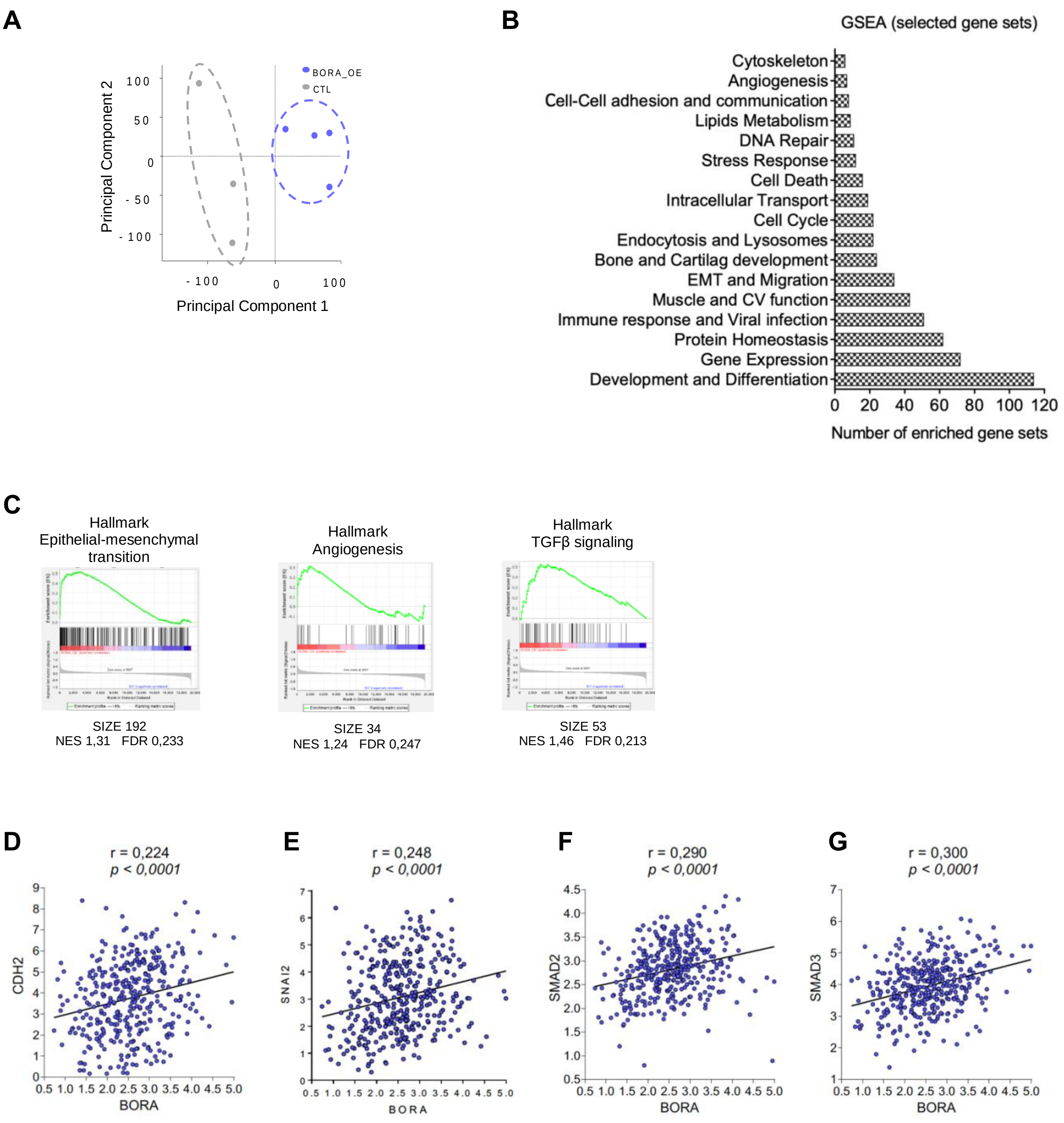


**Supplementary Figure 3. A.** Principal component analysis (PCA) comparing the expression profiles of BORA_OE (n=4) and CTL (n=3) tumors. **B.** Gene set enrichment analysis using the GSEA software. Principal collections of hallmarks, GO gene sets and KEGG pathways were downloaded from the Human Molecular Signatures Database (MSigDB) and used for the GSEA. Graph represents the total number of enriched gene sets related to each process. **C.** Representative GSEA curves of statistically significant enriched gene sets (FDR < 0.5 or p value <0.05) of EMT, angiogenesis and TGFβ signaling. Vertical bars in the red area indicate overexpressed query genes and vertical bars in the blue area indicate underexpressed query genes. **D-G**. Pearson correlation between BORA mRNA levels and mRNA expression of the EMT-related genes: (**D**) CDH2, (**E**) SNAI2, (**F**) SMAD2, and **(G)** SMAD3.

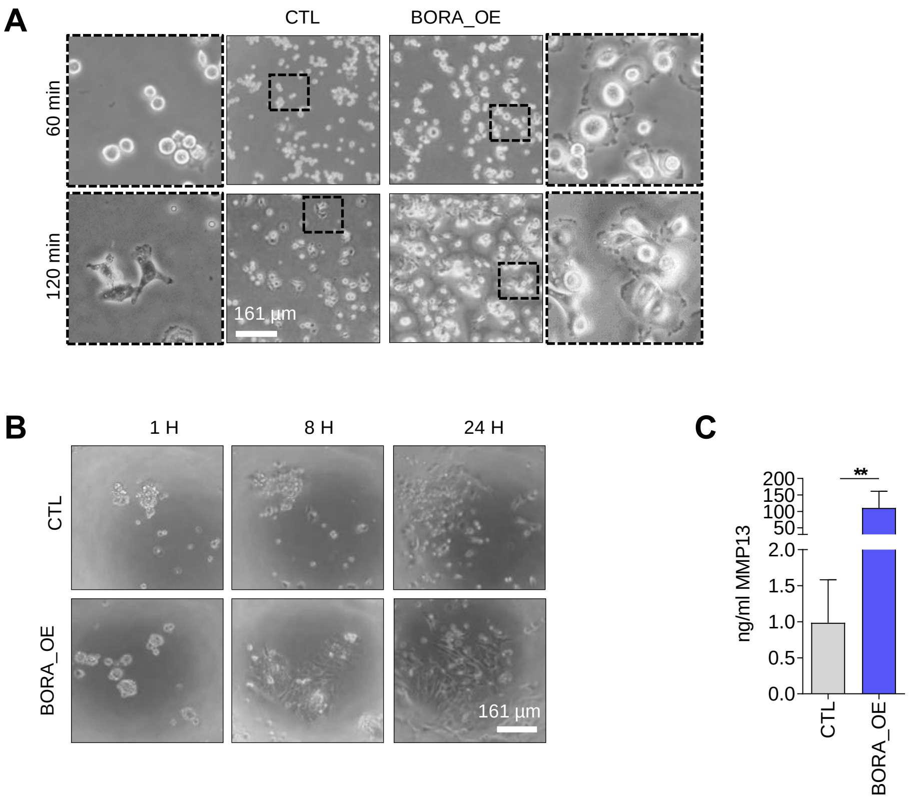


**Supplementary Figure 4. A.** Representative images of SKOV3_CTL and BORA_OE adhered cells in collagen type I coated plates at 60- and 120-min post-seeding. Amplification of selected areas are shown next to each image. **B.** Representative images of disaggregation and dissemination of multicellular aggregates derived from SKOV3_CTL and BORA_OE cells at 1, 8 and 24 h post-seeding. **C.** MMP13 enzymatic activity of SKOV3_BORA_OE cells compared to that of CTL cells. P-values were calculated using two-tailed Student's t-test. *p<0.05; **p<0.01; ***p<0.001, ****p<0.0001. Scale bar: 161 µm.

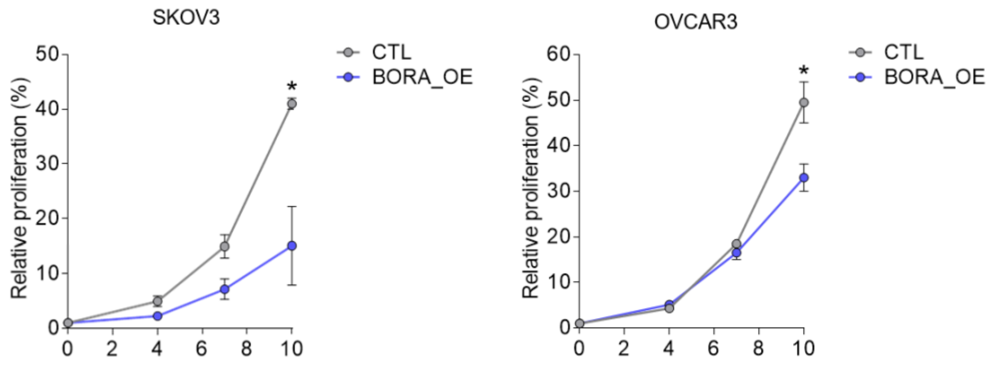


**Supplementary Figure 5. BORA overexpression does not increase proliferation *in vitro*.** Proliferation 10 days time-course of SKOV3 and OVCAR3 BORA_OE cells and CTL cells. Cells were counted every 2 days and relativized to day 0. *P*-values were calculated using two-tailed Student's *t*-test. *p<0.05; **p<0.01; ***p<0.001, ****p<0.0001.

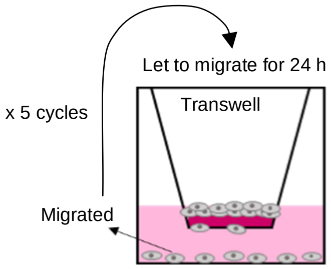


**Supplementary Figure 6.** Scheme of the experimental design to obtain a population of SKOV3 and OVCAR3 highly migrative cells.

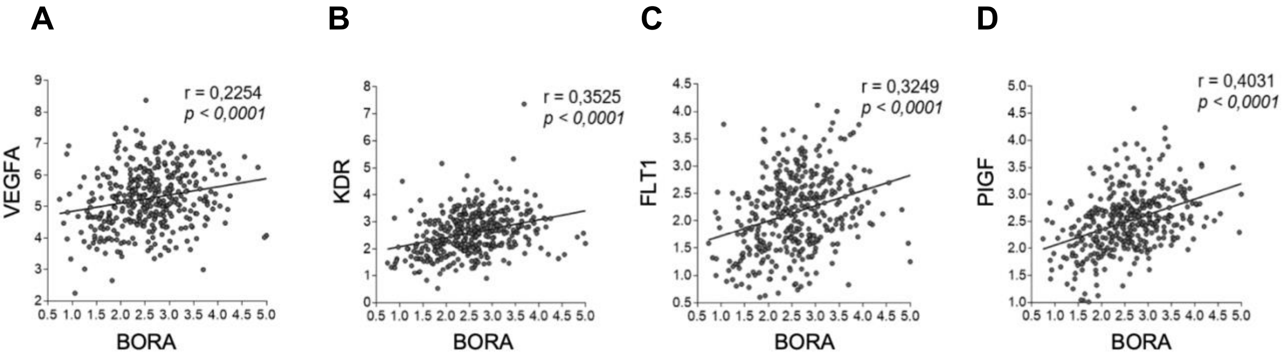


**Supplementary Figure 7.** Pearson correlation between BORA and VEGFA (**A**), KDR **(B)**, FLT1 (**C**), and PIGF (**D**), using data from the Tumor Ovarian Serous Cystadenocarcinoma TCGA cohort (n=381; 2022-v32

## Supplementary tables

**Supplementary Table 1.** General characteristics of the human OC cell lines

| Name | Tumor Type | Source | Medium | Monolayer morphology | Doubling time |
|---|---|---|---|---|---|
| SKOV3 | Ovarian adenocarcinoma | Ascites | McCoy's 5A (Biowest) | Intermediate Mesenchymal | ~ 48 h |
| OVCAR3 | HGSC | Ovary | RPMI (Biowest) | Epithelial | ~ 34 h |
| OVCAR8 | HGSC | Ovary | RPMI (Biowest) | Epithelial | ~ 28 h |

Abbreviations: HGSC, high grade serous carcinoma

**Supplementary Table 2.** Primer sequences used for RT-qPCR

| Gene Name | Primer sequence (5' to 3') | Amplicon length |
|---|---|---|
| BORA | Fw: CCTTGTGAAAGCAGTAACATTCAG<br>Rv: TGTGCATCACTTTCTTTGCAGT | 113 nt |
| CDH2 | Fw: CTCCATGTGCCGGATAGC<br>Rv: CGATTTCACCAGAAGCCTCTAC | 92 nt |
| CDH1 | Fw: GCCGAGAGCTACACGTTCA<br>Rv: GACCGGTGCAATCTTCAAA | 88 nt |
| SLUG | Fw: TGGTTGCTTCAAGGACACAT<br>Rv: GCAAATGCTCTGTTGCAGTG | 77 nt |
| SNAIL | Fw: GCGAGCTGCAGGACTCTAAT<br>Rv: CGGTGGGGTTGAGGATCT | 102 nt |
| CTNNB1 | Fw: GGCTTGGTGAAATAGCAAACA<br>Rv: GTCCAACTCCATCAAATCAGTG | 114 nt |
| MMP10 | Fw: TGGACAGAAGATGCATCAGG<br>Rv: CTTCAGTGTTGGCTGAGTGAA | 94 nt |
| MMP13 | Fw: CCAGTCTCCGAGGAGAAACA<br>Rv: AAAAACAGCTCCGCATCAAC | 85 nt |
| ITGA5 | Fw: CCCATTGAATTTGACAGCAA<br>Rv: TGCAAGGACTTGTACTCCACA | 92 nt |
| COL4A1 | Fw: GGCATGCCTGGTATTGGT<br>Rv: AGGCCCCATATCACCCTTAG | 60 nt |
| COL5A2 | Fw: ACCAGGTGTTCCTGGTCAAC<br>Rv: CAACCCAGCCATTTGAGC | 106 nt |
| COL12A1 | Fw: CTCCCACCATTCCACCAG<br>Rv: CAAAGCCTCCCACAGTATTGA | 141 nt |
| TGFβ | Fw: CCGCTCTTTTTGGCTACCT<br>Rv: AAGAGGGCAGGCAACTCC | 61 nt |
| SRF | Fw: AGCACAGACCTCACGCAGA<br>Rv: GTTGTGGGCACGGATGAC | 80 nt |
| ADAM9 | Fw: GAGTGTGCATATGGTGACTGTTG<br>Rv: ACTGGTTTTTCCTCGGCATA | 67 nt |
| SMAD3 | Fw: CAGCCTTTTGCAGAACACAGT<br>Rv: CAGCAGCAAAGAATAAAGTACCAA | 66 nt |
| RHOJ | Fw: CCTCCACAATGTGTGACATAGAA<br>Rv: TCCTTACTGGGAGCTTTTGC | 65 nt |
| LAMA1 | Fw: TGACCTCCATTCTGACTTACACA<br>Rv: CCTTCCTTACATGGGCACTG | 60 nt |
| ZEB1 | Fw: TGCAGTTTTCAAAGTTAGGAACAA<br>Rv: TGTTGCTCTCTGAGTCATTAAGGT | 74 nt |
| TBP | Fw: GAACATCATGGATCAGAACAACA<br>Rv: ATAGGGATTCCGGGAGTCAT | 87 nt |

Abbreviations: Fw, forward; Rv, reverse; nt, nucleotides

**Supplementary Table 3**. Plasmids and vectors used in this project.

| Vector | Description | Supplier |
|---|---|---|
| pDONR CDS BORA | Coding sequence of human BORA cloned | Dr. Erich Nigg (Basel, Switzerland) |
| pINDUCER20 | Tet-inducible lentiviral vector for ORF expression | Addgene plasmid #44012 |
| pmCherry-Luc | Reporter vector expressing mCherry and Luciferase | Dr. Miguel Segura (VHIR, Spain) |
| psPAX2 | 2nd generation lentiviral packaging plasmid | Addgene plasmid #12259 |
| pMD2.G | 2nd generation lentiviral envelope plasmid | Addgene plasmid #12260 |

**Supplementary Table 4**. siRNAs used in this project.

| Gene | siRNA | Sequence | Supplier |
|---|---|---|---|
| BORA | siRNA BORA<br>siRNA BORA_as | UAACUAGUCCUUCGCCUAUUU[dT][dT]<br>AAAUAGGCGAAGGACUAGUUA[dT][dT] | Sigma-Aldrich |
| CONTROL | siRNA CTL<br>siRNA CTL_as | GUAAGACACGACUUAUCGC[dT][dT]<br>GCGAUAAGUCGUGUCUUAC[dT][dT] | Sigma-Aldrich |
